# Free Will, Freedom of Choice and Frontotemporal Lobar Degeneration***

**DOI:** 10.4103/0973-1229.77440

**Published:** 2011

**Authors:** Daniel A. Drubach, Alejandro A. Rabinstein, Jennifer Molano

**Affiliations:** * *Department of Neurology, Mayo Clinic, 200 First Street SW, Rochester, Minnesota 55905*; ** *Department of Neurology, University of Cincinnati, Ohio*; *** *Revised and peer reviewed version of a Paper read at an International Seminar on Mind, Brain, and Consciousness, Thane College Campus, Thane, India, January 13-15, 2010.*

**Keywords:** *Behavioural variant*, *Free will*, *Freedom of choice*, *Frontotemporal dementia*, *Volitional movement*, *Frontotemporal lobar degeneration*, *FTLD*, *BVFTLD*

## Abstract

The question whether human beings have free will has been debated by philosophers and theologians for thousands of years. More recently, neuroscientists have applied novel concepts and tools in neuroscience to address this question. We submit that human beings do have free will and the physiological substrate for its exercise is contained within neural networks. We discuss the potential neurobiology of free will by exploring volitionally initiated motor activity and the behavioural-response to a stimulus-response paradigm. We also submit that the exercise of free will can be affected in patients with the certain neurological disorders such as the behavioural variant of frontotemporal dementia. Clinicopathological correlation in patients with this disorder provides an opportunity to further elucidate the neural substrate for this fundamental human attribute. We also discuss the clinical correlates of the loss of free will in this population, which is a source of significant distress to patients, significant others and care givers.

## Introduction

The question whether human beings have free will and freedom of choice has been debated for thousands of years by countless philosophers and theologians. More recently, neuroscientists have joined the quest for an answer to this most fundamental of humanity’s questions. It is beyond the scope of this paper to review the infinite number of arguments, both affirming and denying the existence of free will proposed by various individuals throughout the ages. In fact, we believe that the question that must be asked is not whether human beings have or do not have free will, but rather how much free will is present within the confines established by the human beings, and most importantly the individual’s particular condition (which we will refer to as relative free will).

This being said, we argue that the mere fact that human beings question whether free will exists is relative proof of its existence; this logic is along the lines of Descartes postulate “I think, therefore I am.” As a starting premise for this paper, the authors do believe that relative free will is indeed a human attribute and that, as with all aspects of human cognition, it has a neurobiological basis. Furthermore, the authors believe that relative free will and freedom of choice are highly dependent on intact neurological function. In addition, certain neurological disorders that cause damage to specific areas of the brain can limit an individual’s ability to exercise free will.

### Frontotemporal lobar degeneration (FTLD) and its behavioural variant (BVFTLD)

One of the essential components of free will is being able to voluntarily initiate actions (defined as any activity that results in a change in the external and internal universe) as well as modulate and choose from amongst a number of potential responses to internally or externally generated stimuli. Frontotemporal lobar degeneration (FTLD) is a neurodegenerative disorder which causes a number of relatively distinct clinical syndromes, although the underlying neuropathology can be quite variable (for review, see Cairns *et al*., 2007). Patients with the “behavioural variant” of this disorder (BVFTLD) display significant alteration in cognition, behaviour and personality (Neary *et al*., 1998). Although there is significant heterogeneity in the pathological findings, some authors propose that specific neuropathological processes correlate with specific clinical syndromes. For example, Grossman (Grossman *et al*., 2007) has noted that patients with tau-negative FTD have greater social, language and verbally mediated executive function. Hu *et al*. (2007) reported that patients with behavioural-variant frontotemporal dementia with tau-positive pathology were more likely to display poor planning and/or judgment, whereas tau-negative patients displayed impaired personal conduct and a paucity of dysexecutive symptoms. However, the greater evidence seems to indicate that the clinical phenotype correlates with the anatomical distribution of the pathology. Structural imaging studies in patients with BVFTLD have suggested predilection for involvement of bilateral orbital frontal, insular and anterior cingulate cortex (ACC), as well as the right dorsolateral frontal and left premotor cortex (Rosen *et al*., 2002), although the degree and area of involvement may vary by severity of disease (Perry *et al*., 2006; Seeley *et al*., 2008).

Disinhibition, impulsivity and altered goal-oriented action generation, implementation and retro-assessment are prominent features of this syndrome. Furthermore, patients with BVFTLD frequently display a rigid and predictable response in situations evoking the stimulus-response paradigm. We argue that FTLD provides an example of a neurological disorder that interferes with an individual’s exercise of free will and freedom of choice. Furthermore, clinicopathological correlation in this population can help clarify the neurological substrate for this most human of attributes.

### Free will and BVFTLD

The exploration of whether and how free will is affected in patients with neurological disease (such as BVFTLD) has important clinical implication. For example, patients often display behaviours that may be hurtful or offensive to family members which can result in social discord. Furthermore, family members and caregivers frequently hold the patient responsible for those behaviours. A recent example encountered by one of the authors involved a “normally” faithful and devoted husband who had a number of extramarital affairs and displayed sexually related inappropriate behaviour after developing BVFTLD. In counselling caregivers, the clinician must make an assessment of how much control the patient has over his or her own behaviour and how much “responsibility” to attribute to the patient. The author has found that a comprehensive discussion with the patient and caregivers regarding these issues may help alleviate destructive tensions aroused in the patient’s immediate social environment caused by his or her behaviour.

## Definition of Free Will

Our first and perhaps most demanding of challenges is the definition of free will. The numbers of perspectives that can be utilised to accomplish this task are numerous. However, for purposes of this discussion, we will limit our discussion of free will as it relates to physical actions (rather than including other aspects as thoughts, emotions, etc.). Furthermore, we will approach the subject of free will extracting from a framework utilised by the fifteenth century philosopher Benedictus Spinoza. Although Spinoza’s theory on pantheism did not allow for free will in human beings, he did propose a model which is useful for our discussion. In his magnificent work “*Ethics*,” Spinoza states the following: “That thing is called ‘free’ which exists solely by the necessity of its own nature, and in which its own nature alone is the cause of its actions. On the other hand, that thing is necessary, or rather constrained, which is determined to a fixed and definite method of existence or action by something external to itself” [Part I (on God) definition 7 (Spinoza and Parkinson, 2000)]. Therefore, Spinoza would argue that we exercise freedom when we are the “cause” of our actions.

To illustrate this concept, we will utilize the example frequently alluded to by students of Spinoza’s works. Paul and Peter are walking on a narrow bridge; if Paul pushes Peter off the bridge, Paul is the cause of Peter’s fall and thus he exercised free will. If the wind pushes Paul against Peter and results in Peter’s fall, then it was not Paul but the wind which caused Peter’s fall. Paul did not exercise free will in this situation, as his behaviour was caused by something external to him.

We can expand and apply this concept to neurophysiology by the following example. If we were to utilise transcranial magnetic simulation to stimulate the motor cortex of a subject, we could easily induce movement of a limb. In this case, the subject was not the cause of the particular movement (the cause was the electromagnetic field) and he or she consequently did not exercise freedom of will. In contrast, if the subject voluntarily produced the movement, then he or she would be the cause of the movement and thus would have exercised free will.

Even more, let us translate this further to a neurobiological model. If a neuron or population of neurons depolarize spontaneously, and their depolarization is not caused by inhibitory and/or excitatory signals from other neurons, then those depolarizing neurons are the cause of their own depolarization. Instead, if the depolarization is wholly caused by the influence of additive excitatory and inhibitory signals which stem from other neurons, then the depolarizing neurons are not the cause of their own depolarization. A third scenario is also plausible. If that same neuron, or population of neurons, receives signals from other neurons which influence, but not totally cause their depolarization, then we can deduce that the depolarizing neuron/s are a “partial” cause of their own depolarization. This is, we propose, the most plausible explanation for voluntary initiation of movement. However, it is a hypothesis that is yet to be proven.

We will approach our discourse on free will and its neurological substrate from the following two perspectives: the wilful initiation of motor acts and the ability to choose among different options in response to an identifiable stimulus. We will then discuss BVFTD as a model of a neurological disorder which can affect the exercise of one or both of these functions.

## The Neurobiology of Volitional Movement

From a neural perspective, movement is caused by depolarization of brainstem (for the cranial musculature) or spinal (for muscles in the trunk and limbs) motor neurons. These motor neurons receive signals from neurons in the primary motor cortex, which sends their axons “downstream” along the corticospinal tract to the alpha motor neurons. Other than the poorly understood depolarization of single or small number of neurons resulting in a degree of “background noise,” spontaneous synchronous activation of a large population of neurons in the primary cortex (which would result in movement) is not known to occur under normal conditions. In contrast, epilepsy is an example of an abnormal state that results in spontaneous primary cortex activations and corresponding motor activity.

Instead, the primary cortex becomes active upon receiving inputs from cortical neurons “upstream” in the “motor hierarchy.” The cortical motor system is organised in a hierarchical manner at the “bottom” of which lies the primary cortex. Components of this hierarchy include the posterior parietal, premotor, presupplementary, supplementary and cingulate cortex, as well as subcortical regions. These areas are not only connected serially but also in parallel. Within this hierarchy, there are groups of neurons that become active with various steps in the movement process, including the imagination of motor acts, the intention to move, the refrain of movement, the performance of movement and so forth.

The hierarchical organisation of the cortical motor system implies that there are higher order association cortical areas that send “top down” connections to the primary motor cortex. With regard to the neurobiology of volitional movement, several questions must be considered. For example, at what level of this hierarchy does volition act? Reason would lead us to believe that it should be at the top. Thus, if the primary motor cortex were “isolated” from top down connections, voluntary movement would not be possible. But even more importantly, how is it that volition as an “initiator of neural activity” interacts with neuronal elements to bring about the simultaneous and synchronous firing of the large population of neurons required for even the simplest of movement? After all, volition is a metaphysical entity; it cannot be expressed or measured through physical means. Therefore, attempting to explain the relationship between volition and neuronal activity evokes the eternal and yet to be satisfactorily deciphered mind-body problem.

To partially address this question, we can conceptualise the existence of a population of “source” neurons located at the height of the motor hierarchy, which act as the initiators of impulses travelling down this hierarchy to the primary motor cortex. How is it that those source neurons depolarise, initiating the movement cascade? There are a number of potential scenarios. The first is that volition as a force in a different dimension and “outside” of the brain somehow activates the neurons, as does TMS in the example above. This notion is very much contained within religious thought, which states that the “soul” is outside of the body and somehow drives it to accomplish its mission. The other possibility is that volition is a property contained within the source neurons, which possess the potential to depolarise as “they choose.” This notion is akin to Aristotle’s discussion of the unmoved mover. Aristotle argued that there must logically be a first, unmoved mover in order to explain all other motion; motion cannot begin without the prior existence of something to impart motion to another thing (Barnes, 1995). His unmoved mover does not itself move, but contains within itself the source of all motion. In order to create an analogy for our discussion, we need to explain volitional movement (as proposed in the second scenario) on the basis of a neuron, or population of neurons, which itself does not require to be depolarised by other neurons, but is capable of depolarizing other neurons downstream.

However, it would seem that a third scenario, which incorporates features from the first two, is the most plausible theory to explain voluntary movement. Let us conceive a “volitional system” comprised a population of neurons which receive inputs from multiple other neurons distributed throughout the entire central nervous system. These inputs convey information about a person’s specific needs and wants, personal and social norms for behaviour, current environmental status, memories about effectiveness and consequences of past behaviour as well as a large body of additional information. The volitional system integrates this information and “comes up” with a variant plan which includes the many dimensions of an act, including whether to act or not, as well as the what, when and how aspects of an action. Therefore, utilising the definitional scheme described above, the volitional system is a “partial” cause of its actions; it contains some “autonomy” but also incorporates “information external to itself.”

The potential structural candidates within the brain for this system are many. For example, the posterior parietal cortex has been shown to be involved in action selection and movement preparation in monkeys (Cui and Andersen, 2007). The left dorsolateral prefrontal cortex (Brodmann’s area 9) has also been implicated (MacDonald *et al*., 2000). However, it is likely that components of this system are multiple and further studies are needed to better localise and characterise them.

A discussion on voluntary initiation of motor activity would not be complete without a brief exploration on the relationship between consciousness (or rather awareness) and volition. The extensive scientific debate on this issue was perhaps sparked by a landmark paper published by Libet *et al*. (Libet *et al*., 1983). It is beyond the scope of this paper to discuss at length this study and the hundreds of subsequent publications arguing for and against its findings and conclusions, but a brief description seems fitting. Libet recorded a number of parameters including the timing of the “readiness potential” (RP), the time of perceived intention to move (W) and time of movement as measured by EMG in volunteers instructed to move their writs at a time of their choosing. Libet found that RP (an unconscious neurophysiological event) preceded W by approximately 300 to 500 milliseconds. The author thus concluded that “cerebral initiation of a spontaneous, freely voluntary act can begin unconsciously, that is, before there is any (at least recallable) subjective awareness that a ‘decision’ to act has already been initiated cerebrally.” Most recently, Soon *et al*. (2008) utilised fMRI to demonstrate that activation of the frontopolar and parietal cortex that occurred 10 seconds before awareness of intent predicted the nature of a “volitional” decision. Hallet (2007) recently wrote an excellent review on the neurophysiology of volitional movement and concluded that freedom of will is a “perception” rather than an “initiating force.” That is, a person believes him or herself to be free because he or she is aware of the intent to move, but that intent is generated at an unconscious level and only reaches awareness at a later stage.

The fact that volitional movement may be initiated unconsciously has brought into question the existence of free will, but in the authors’ opinion, the matter is far from clear. The line separating conscious from unconscious cognitive processes is probably not as “rigid” as suggested by psychoanalytic theory, one of its first proponents. Instead, this line may be quite fluid, allowing cognitive activity to constantly transition between both states. We suggest that volitional activity may arise from a combination of both conscious and unconscious cognitive processes.

### Neurological disease and volitional initiation of movement

Some light into the neurobiological substrate for volitional movement can be shed by the study of neurological syndromes that specifically interfere with the initiation of volitional activity. Such is the case of “abulia” (from the Greek “lack of will”), a syndrome characterised by lack of spontaneity of action or speech, deficiency in initiation, apathy, inertia, mental slowness, reduction in excursion of motion, paucity of motivation, poor attention and easy distractibility (Vijayaraghavan *et al*., 2002).

Patients with BVFTLD frequently display variable degrees of abulia. Patients may become reclusive and fail to initiate or participate in social, recreational or vocational activities. Some patients fail to initiate conversations. Family members frequently complain that if “left to his or her self” the patient “just sits around the house.” In advanced stages, the patient may fail to initiate or participate in self, home and family care. The authors have also seen extreme cases of inactivity in patients with advanced BVFTLD where the term Akinetic mutism can be applied. This refers to a syndrome, initially described in a patient with a third ventricle cyst by Cairns (1941) and characterized by lack of initiation of any type of motor activity, including speech. The affected patient remains motionless and mute, although seemingly aware of the surrounding environment. Lack of activity initiation can be a significant source of distress and frustration in those caring for patients with BVFTLD and it is very much a source of increased burden of care.

If we aim at clinicopathological correlation, we can presume that brain areas involved in patients with BVFTLD have a role in either the volitional initiation of actions or in the transport of impulses from the “volitional source system” to the primary motor areas. However, considering such factors as the relatively large structural involvement, the fact that functional involvement as documented by functional neuroimaging (PET, SPECT and fMRI) can exceed structural involvement, as well as other factors, it is difficult at this time to further identify the structures responsible for voluntary action initiation.

Of course, even being able to estimate the localisation of the volition-neuronal interaction would not explain the mechanism for that interaction. Still, we submit that disorders of motor initiation are an example of neurological conditions that interfere with an individual’s voluntary initiation of movement, an important component of free will.

## The Neurobiology of Freedom of Choice

The stimulus-response paradigm proposes the existence of a “black box” that determines the behavioural, cognitive and emotional response to an extrinsically or intrinsically generated stimulus. In order to perform this task, the black box utilises a set of cognitive functions grouped together within the “umbrella” term of “executive functions.” The definition of executive functions varies among investigators, but the term is a metaphor for the concept of an executive within a business or administrative setting that manages employees and systems to achieve a specific goal. Thus, within a cognitive model, the concept refers to an executive cognitive system that manages other aspects of cognition to “appropriately” achieve goal-oriented behaviour. Executive functions include initiating, planning and sequencing behaviours (taking into account past experience and current knowledge), altering behavioural and affective responses according to ongoing changes in circumstances as well as monitoring and regulating thought, affect and behaviour.

The brain areas most frequently implicated in control of executive behaviour include, among others, the dorsolateral prefrontal cortex (DLPFC) and the ACC (Devinsky *et al*., 1995; Goldman-Rakic, 1987; Lie *et al*., 2006; Pardo *et al*., 1990; see also, MacDonald *et al*., 2000), although the role of each continues to be a source of debate.

### Automatic response and stimulus bound

A fundamental aspect of the executive functions contained within this “black box” is the ability to suppress the “automatic response” to a stimulus. The automatic response refers to a reaction that fails to take into account situation-specific circumstances that may make such response inappropriate. An example may clarify this concept. A patient with BVFTLD became involved in a fight after another individual called him “shorty.” The patient failed to incorporate into his decision to attack the fact that the offending individual was joking and much larger and stronger than the patient, resulting in unfavourable results for the patient.

The term “stimulus bound” has been utilised to denote a state where an individual cannot inhibit or dissociate from the automatic response to a stimulus. The response is determined solely by the characteristics of the stimulus rather than by the associated circumstances. Clinical correlates of stimulus-bound behaviour displayed by patients with BVFTLD include impulsiveness, poor tolerance to frustration, angry responses to minor provocation, among others.

A variant of stimulus-bound behaviour is utilisation behaviour. Patients with this disorder cannot suppress the urge to utilise an object in the immediate environment. As described above, it is the stimulus, rather than the circumstances, which guides the response. Such an individual is at “the mercy” of the environment. Examples abound in patients with FTDLV; family members report the necessity to hide food, since a patient with this behaviour will “eat what he or she sees.” A patient evaluated by one of the authors could not “resist the urge” to utilise the medical examination tools placed in a table a short distance away. He put on the author’s stethoscope, tapped the desk with the reflex hammer and even picked up the telephone receiver in the office and proceeded to make a phone call. Another patient read everything readable in his field of vision (such as street signs or advertisements).

Suppression of the automatic response (which is lacking in stimulus-bound and utilisation behaviour) is a fundamental premise in order to embark on any subsequent response. Without this suppression, any potential ulterior choice is rendered difficult or impossible. Thus, an individual who succumbs to the automatic response frequently loses the freedom to respond otherwise. This is because, in order to exercise freedom of choice, at least two options must be available to choose from. If the automatic response is the only one available, then freedom of choice is not attainable.

In fact, some authors have proposed that human freedom is not expressed by action generation but rather by modulating the response to a stimulus which is generated unconsciously. Libet (mentioned in previous paragraphs) suggested that human freedom may be expressed by “free won’t” (Libet, 1999); although (as discussed above) actions may be initiated subconsciously, an individual retains the conscious decision as to how to respond to that “impulse.” This reasoning is in line with the philosopher John Locke’s position on voluntary behaviour and free will: he argued that the feature defining voluntary behaviour is the ability to “*postpone* a decision long enough to reflect or deliberate upon the consequences of a choice” (Locke and Wilburn, 1947).

Indeed, recent functional MRI studies suggest that the brain contains a network for inhibiting intended actions. Brass and Haggard (2007) demonstrated strong activation of specific areas of the frontomedian cortex when an individual prepares a manual of actions but then intentionally cancels its performance, as compared with preparation and completion of that same action. Since these areas are frequently involved in BVFTLD, it is possible that this may play a part in the deficiency of action suppression displayed by patients with this disorder.

Thus, the stimulus-response paradigm can be used to propose a possible schematic explanation for the freedom of choice. With a particular internally or externally generated stimulus, one must have an intact “black box” in order to have freedom of choice. According to the prior research mentioned previously, the components of this “black box” may include the dorsolateral prefrontal cortex, the anterior cingulate and the frontomedian cortex. An intact “black box” allows for intact executive function, leading to the ability to suppress an automatic cognitive, behavioural and emotional response and to choose a more appropriate one. When this “black box” is compromised, as it is with BVFTLD, executive function is impaired, leading to an inappropriate cognitive, behavioural and emotional response due to the inability to suppress the automatic one. However, more research should be performed in order to see if this proposed schematic holds true.

## Conclusions [see also Figure 1]

**Figure 1:**
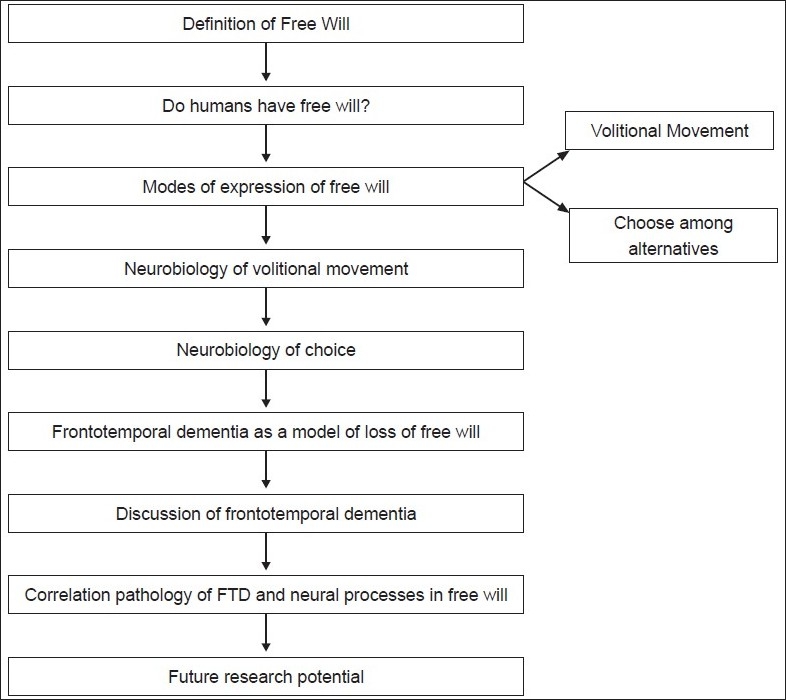
Flowchart of paper

In this paper, we have argued that free will and freedom of choice are human attributes that have a neural substrate, although the location and characteristics of that substrate remain to be better delineated. Consequently, damage to specific areas of the brain such as those that occur in patients with BVFTLD can interfere with the full exercise of this human trait. This brings up a unique dimension to the clinical care of these patients, including the attribution of responsibility for socially inappropriate behaviour. Further advances in the understanding of clinicopathological correlates of patients with BVFTLD may also help characterise the neural mechanisms for free will and freedom of choice. Furthermore, correlating “physical attributes of brain function” with “existential issues such as freedom of choice” will, in our opinion, help in further clarifying the “mind-body” problem.

### Take home message

For centuries, philosophers, theologians and psychologists have argued whether man has free will. Since the brain is the “source” of human physical, emotional and cognitive activity, it seems reasonable to tackle this issue using a neuroscientific approach. We argue that free will is compromised in patients with certain neurological conditions, and especially those with the behavioural variant of frontotemporal dementia. By correlating behavioural expressions of loss of free will with the type and location of neurological damage, we could potentially identify brain processes that are responsible for the expression of this most sublime of human attributes. This may shed some more light on the century-old argument of whether man has free will.

## Questions That This Paper Raises

1. What are the neurobiological mechanisms that determine the timing and characteristics of neural activity that results in goal-oriented voluntary movement?
2. Can rehabilitation strategies be useful in modulating the stimulus-bound behaviour in patients with BVFTD?
3. Does the type of neuropathology rather than its localisation determine the clinical manifestations in patients with BVFTD?
4. How much responsibility for their actions can be ascribed to patients with BVTFD?

## About the Author

Dr. Daniel Drubach was born in Buenos Aires, Argentina, and grew up in California. He completed postgraduate medical training at the University of Maryland and is board certified in both Neurology and Psychiatry. His areas of expertise include traumatic brain injury and coma after neurological injury. He was Associate Professor of Neurology and the director of the Traumatic Brain Injury and the Coma programme at Kernan Hospital and the University of Maryland for several years, and is currently Consultant in the Departments of Neurology and Psychiatry at Mayo Foundation and Clinic in Rochester, Minnesota. He is the author of a popular book on neurosciences and is also a fiction author, with short stories published in the Hispanic Culture Review and others.

## About the Author

Dr. Alejandro A. Rabinstein was born in Cordoba, Argentina, where he earned his medical degree and completed postgraduate training in Internal Medicine. He then moved to the United States where he trained in Neurology at University of Miami. Dr. Rabinstein is a board-certified neurologist and his areas of expertise include neurocritical care and cerebrovascular diseases. He is currently Associate Professor of Neurology at Mayo Clinic, Rochester. Beyond Medicine, his interests include literature, philosophy and the history of religion.

## About the Author

Dr. Jennifer Rose V. Molano is a board-certified neurologist who received a Bachelor of Science degree in Psychology and Latin from the University of Michigan at Ann Arbor. After returning to her home state for both medical school and neurology residency training at West Virginia University, she completed fellowships in behavioural neurology at the Mayo Clinic in Rochester, Minnesota and sleep medicine at Vanderbilt University in Nashville, Tennessee. She is currently an Assistant Professor with neurology department at the University of Cincinnati in Ohio. In addition to investigating the interface between cognition and sleep, her interests include patient and medical education. She also enjoys running, and playing the piano.
